# Digital Orthodontic Setups in Orthognathic Surgery: Evaluating Predictability and Precision of the Workflow in Surgical Planning

**DOI:** 10.3390/jcm14155270

**Published:** 2025-07-25

**Authors:** Olivier de Waard, Frank Baan, Robin Bruggink, Ewald M. Bronkhorst, Anne Marie Kuijpers-Jagtman, Edwin M. Ongkosuwito

**Affiliations:** 1 Department of Dentistry—Orthodontics and Craniofacial Biology, Radboud University Medical Center, 6500 HB Nijmegen, The Netherlands; frank.baan@radboudumc.nl (F.B.); robin.bruggink@radboudumc.nl (R.B.); edwin.ongkosuwito@radboudumc.nl (E.M.O.); 2 Radboudumc 3DLab, Radboud Institute for Health Sciences, Radboud University Medical Center, 6500 HB Nijmegen, The Netherlands; 3 Department of Dentistry, Radboud Institute for Health Sciences, Radboud University Medical Center, 6500 HB Nijmegen, The Netherlands; ewald.bronkhorst@radboudumc.nl; 4 Department of Orthodontics, University Medical Center Groningen, University of Groningen, 9713 GZ Groningen, The Netherlands; a.m.kuijpers-jagtman@umcg.nl; 5 Department of Orthodontics and Dentofacial Orthopedics, School of Dental Medicine, Medical Faculty, University of Bern, CH-3010 Bern, Switzerland; 6 Faculty of Dentistry, Universitas Indonesia, Jakarta 10430, Indonesia

**Keywords:** orthodontics, CBCT, orthognathic surgery, digital setup

## Abstract

**Background:** Inadequate presurgical planning is a key contributor to suboptimal outcomes in orthognathic surgery. This study aims to assess the accuracy of a digital surgical planning workflow conducted prior to any orthodontic intervention. **Methods:** Digital planning was performed for 26 patients before orthodontic treatment (T0) and compared to the actual preoperative planning (T1). Digitized plaster casts were merged with CBCT data and converted to orthodontic setups to create a 3D virtual head model. After voxel-based registration of T0 and T1, dental arches were virtually osteotomized and repositioned according to planned outcomes. These T0 segments were then aligned with T1 planning using bony landmarks of the maxilla. Anatomical landmarks were used to construct virtual triangles on maxillary and mandibular segments, enabling assessment of positional and orientational differences. Transformations between T0 and T1 were translated into clinically meaningful metrics. **Results:** Significant differences were found between T0 and T1 at the dental level. T1 exhibited a greater clockwise rotation of the dental maxilla (mean: 2.85°) and a leftward translation of the mandibular dental arch (mean: 1.19 mm). In SARME cases, the bony mandible showed larger anti-clockwise roll differences. Pitch variations were also more pronounced in maxillary extraction cases, with both the dental maxilla and bony mandible demonstrating increased clockwise rotations. **Conclusions:** The proposed orthognathic surgical planning workflow shows potential for simulating mandibular outcomes but lacks dental-level accuracy, especially in maxillary anterior torque. While mandibular bony outcome predictions align reasonably with pretreatment planning, notable discrepancies exceed clinically acceptable thresholds. Current accuracy limits routine use; further refinement and validation in larger, homogeneous patient groups are needed to enhance clinical reliability and applicability.

## 1. Introduction

Presurgical orthodontic treatment typically constitutes an integral component of an orthognathic surgery treatment protocol. Its primary objective is to achieve dental arches that occlude properly after surgical correction of the skeletal discrepancy. Particularly, the extent of incisor decompensation obtained during the presurgical orthodontic treatment dictates the range of the jaw repositioning during surgery and is a major factor in determining the success or failure of treatment outcomes [1,2,3,4]. This includes establishment of correct torque, elimination of tooth-size discrepancies, and establishment of congruent dental arch forms during orthodontic treatment, facilitating the establishment of a correct sagittal occlusion after surgery. This might necessitate using Class III elastics in Class II scenarios (and vice versa) and undertaking tooth extractions during orthodontic therapy, thereby enabling the greatest possible surgical correction of the skeletal discrepancy [4,5,6]. While presurgical orthodontic treatment is an essential step in correcting jaw deformities, it has been well-documented that in some cases the “surgery-first” approach also yields stable outcomes and offers several advantages over the conventional approach [7,8].

Conventional orthognathic treatment involves conducting model surgery just before the actual surgical intervention. Historically, several techniques were successfully developed for planning surgical cases using plaster models and cephalograms for planning postsurgical occlusion. Presently, advanced digital techniques using three-dimensional imaging for the comprehensive representation of the facial soft tissue, skeletal configurations, and dentition (comprising a triad) in a 3-dimensional format [9,10,11] are the state-of-the-art approach equipping the surgeon with the tools to perform virtual 3D osteotomies prior to surgery, enabling a precise and predictable correction of the dysgnathia.

Utilizing digitized dental models has facilitated the construction of digital orthodontic setups which are as accurate as manual setups as documented in the literature [12,13]. The introduction of digital orthodontic setups has opened avenues to integrate these setups in the virtual orthognathic surgical planning.

In orthognathic surgery, inadequate planning before the procedure is a significant factor behind unfavorable results or postoperative complications [2,14,15]. Theoretically, pre-planning orthodontic treatment ahead of surgery should reduce alterations in treatment plans and enhance predictability, allowing for more precise patient information disseminated at the start of treatment [16]. Yet, the accuracy of expedited orthognathic planning at the start of treatment remains unverified. Therefore, this study aims to verify whether accurate simulation of surgical planning can be achieved at the start of treatment before any orthodontic intervention with the help of orthodontic setups.

## 2. Materials and Methods

### 2.1. Patients

Data for this study were sourced from the patient records maintained by the Section of Orthodontics and Craniofacial Biology, Department of Dentistry, Radboud University Medical Centre in Nijmegen, the Netherlands. The research was carried out from July 2021 to February 2022. Only patients requiring a combined orthodontic–orthognathic surgical treatment were included. Exclusion criteria were the presence of a developmental deformity, a previous history of orthognathic surgery, ‘surgery-first’ cases, or missing data; thereby, 11 patients were excluded. The final sample included twenty-six consecutive patients. All patient data were de-identified prior to analysis, and all patients gave informed consent. The patients were treated by 8 postgraduates, supervised by two experienced orthodontists in an academic clinical setting. Ethical clearance for this research was granted by the Institutional Review Board at the Radboud University Medical Centre (2016–2690).

### 2.2. Data Acquisition

Two Cone Beam Computed Tomography (CBCT) scans were acquired for each patient: prior to start of orthodontic treatment (T0) and one prior to surgery (T1). CBCT (KaVo 3d eXam, KaVo Dental GmbH, Biberach/Riss, Germany) scans were acquired in the natural head position in extended field modus (voxel size 0.3 mm, field of view 17 × 23 cm, scanning time 17.8 s) and exported into a digital imaging and communications in medicine format. Directly after each CBCT scan, digitized plaster models (R500 3D Dental Laser scanner, 3Shape^®^, Copenhagen, Denmark) from the dental arches were obtained and exported to Standard Tessellation Language (STL) files.

### 2.3. Orthodontic Setup Production

**Figure 1:**
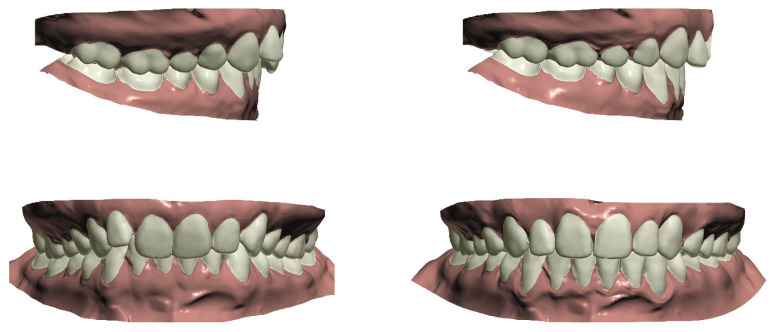
Example of orthodontic setup. Original dental model on the left and orthodontic setup on the right side.

The STL files derived from the digitized plaster casts, along with the DICOM files from the CBCT scans taken at the start of treatment, were imported into Ortho Analyzer™ software (version 2020-1, 3Shape^®^, Copenhagen, Denmark). All dental model setups were prepared once by a single experienced operator (OW). This process included a semi-automated definition of the upper and lower arch forms, tooth axes, rotational points, and segmentation of tooth crowns. Each tooth crown was segmented semi-automatically following the identification of mesial and distal contact points and gingival margins. Subsequently, the segmented teeth were virtually repositioned into their planned decompensated alignment relative to the corresponding CBCT scan to generate a digital orthodontic setup (Figure 1) [17].

Tooth positioning adhered to the treatment plan and fundamental occlusal principles, ensuring proper molar relationships reflecting the postsurgical jaw position, correct crown angulation and inclination, absence of rotations or interdental gaps, an appropriate occlusal plane, accurate interproximal contacts, and normal overjet and overbite (1–4 mm). The original mandibular intercanine distance was preserved and used as a reference for determining the final maxillary arch form and width.

The goal of this setup is to create congruent well-aligned arches, prepared for surgical osteotomy according to the treatment plan [18]. The digital orthodontic setups were exported as STL files.

### 2.4. Surgical Planning

A three-dimensional virtual augmented head model was generated using Maxilim software (version 2.3.0, Medicim NV, Mechelen, Belgium) [19]. Virtual Le Fort I and bilateral sagittal split osteotomies (BSSO) were then executed on the preoperative CBCT scans of patients who had completed their orthodontic preparation. The maxillary and mandibular bony segments were moved to the desired positions in order to create 3D facial harmonization as simulated in all three dimensions by the Maxilim software.

### 2.5. Measuring Differences Between Pretreatment and Presurgical Orthognathic Surgical Planning

To measure the differences between the pretreatment orthognathic surgery treatment planning (T0 planning, before any orthodontic intervention) and presurgical orthognathic planning (T1 planning), the following steps had to be performed in order to align the two CBCT scans and the corresponding digitized plaster casts to integrate the virtual setup in the surgery planning.

#### 2.5.1. Step 1: Incorporation of Orthodontic Setup with CBCT T0

**Figure 2:**
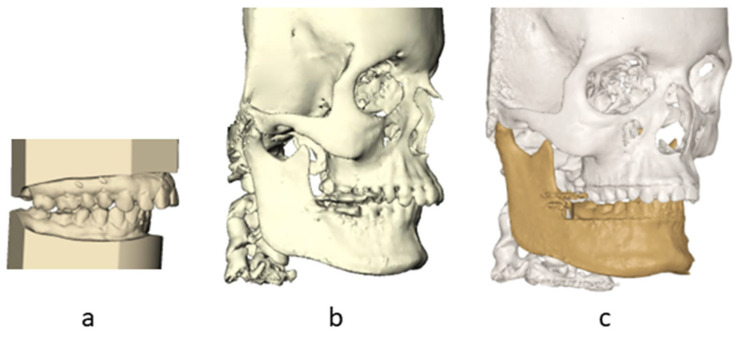
Incorporation of orthodontic setup with CBCT T0. (**a**) orthodontic setup; (**b**) CBCT T0; (**c**) integration of setup in CBCT T0.

IPS CaseDesigner (version 2.2.4, KLS Martin Group, Tuttlingen, Germany) was used to superimpose the digitized plaster casts into the pretreatment CBCT [20]. The 3D-rendered skulls with dentitions and the orthodontic setups were imported into the Maxilim^®^ software. The orthodontic setups were fused with the pretreatment CBCT using the transformation matrices of the fusion of the dental model with the CBCT in IPS Case Designer [20] (Figure 2). All the fusion steps resulted in one pretreatment model consisting of a CBCT with a corresponding orthodontic setup and one preoperative model with a CBCT and corresponding dental model.

#### 2.5.2. Step 2: Registration of CBCT T0 Towards Surgery Planning

**Figure 3:**
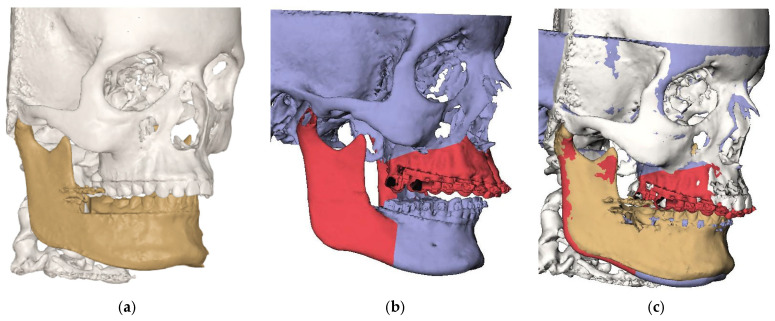
Registration of CBCT T0 towards surgery planning. (**a**) CBCT T0; (**b**) virtual head model T1; (**c**) registration of T0 model towards T1 model.

The preoperative 3D virtual head model (T1) was aligned with the pretreatment planned 3D virtual head model (T2) through voxel-based matching (VBM). For this superimposition, a subvolume comprising the anterior cranial base, which remains unaffected by orthodontic treatment or surgically assisted maxillary expansion (SARME), was utilized (Figure 3).

#### 2.5.3. Step 3: Orthognathic Planning of T0 Model

**Figure 4:**
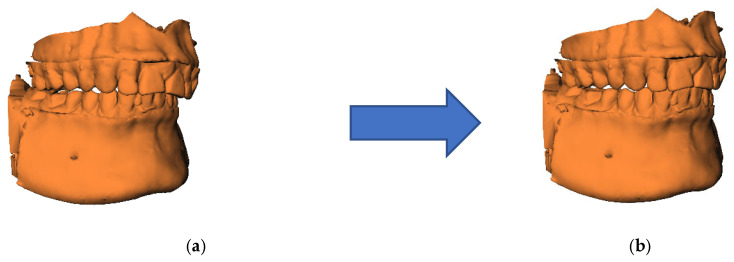
Orthognathic planning of T0 model. T0 model in original occlusion (**a**) and planned occlusion (**b**).

The dental arches of the orthodontic setup were subsequently used to create an occlusion using the virtual occlusion tool in Maxilim [21]. The osteotomized maxilla and mandible were exported as one STL file in the planned occlusion (Figure 4). This step is blinded from the T1 model.

#### 2.5.4. Step 4: Registration of Osteotomized Segments

**Figure 5:**
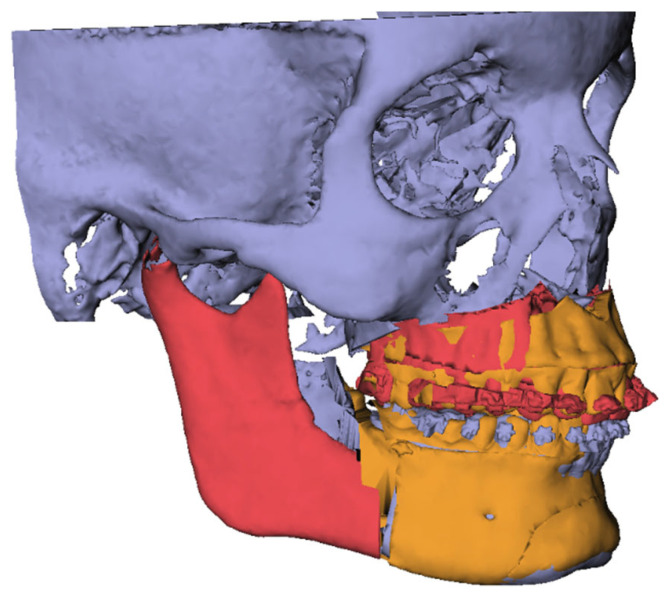
Registration of osteotomized segments.

The pretreatment and preoperative virtually osteotomized maxillary and mandibular bony segments were exported as STL files to 3DMedX software (version 1.1.8, 3D Lab Radboudumc, Nijmegen, The Netherlands). The pretreatment osteotomized segments of the maxilla and mandible were registered to the preoperative planning based on the bony non-dental parts of the maxilla, based on surface-based matching (Figure 5). The preoperatively planned maxilla was used as a landmark to be able to distinguish true maxillary and mandibular dental and mandibular bony differences.

#### 2.5.5. Step 5: Construction of Virtual Triangles

**Figure 6:**
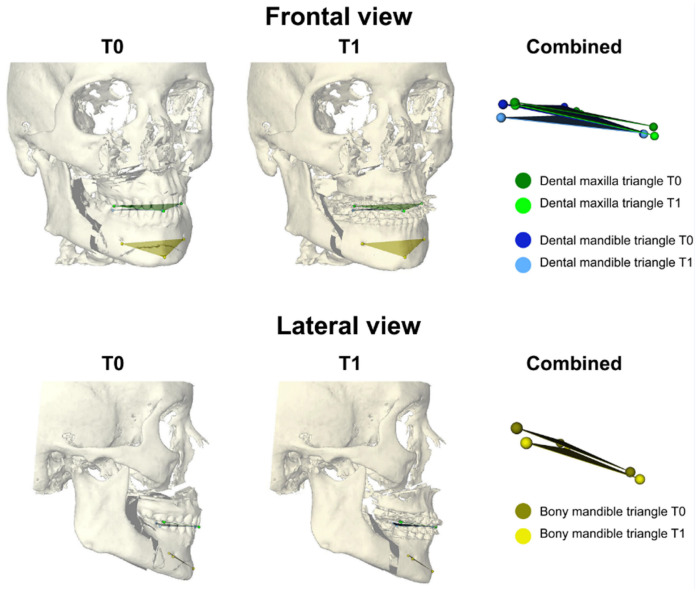
Virtual triangles on T0 and T1 planning. On both planning models (T0 and T1) three triangles (green, blue, and yellow) are obtained to measure dental and bony differences between both models.

To determine the position of the upper and lower arches and the distal mandibular segment, three anatomical landmarks were identified on each of the three segments on the pretreatment and preoperative models. Landmarks were used to create a triangle in order to obtain the 3D position and orientation. On the dental maxilla (Figure 6 green), the mesial buccal cusp of the right first molar (16), upper incisor (UI), and mesial buccal cusp of the left first molar (26) were indicated. On the dental mandible (Figure 6 blue), the mesial buccal cusp of the left first molar (36), lower incisor (LI), and mesial buccal cusp of the right first molar (46) were indicated. On the distal mandibular bony segment (Figure 6 yellow), pogonion (Pog) and left and right mental foramina were identified. The landmarks defined the vertices of a virtual triangle that captured the three-dimensional position and orientation of the bone segments and dental arches (Figure 6).

#### 2.5.6. Step 6: Calculation of Rotational and Translational Movements

**Figure 7:**
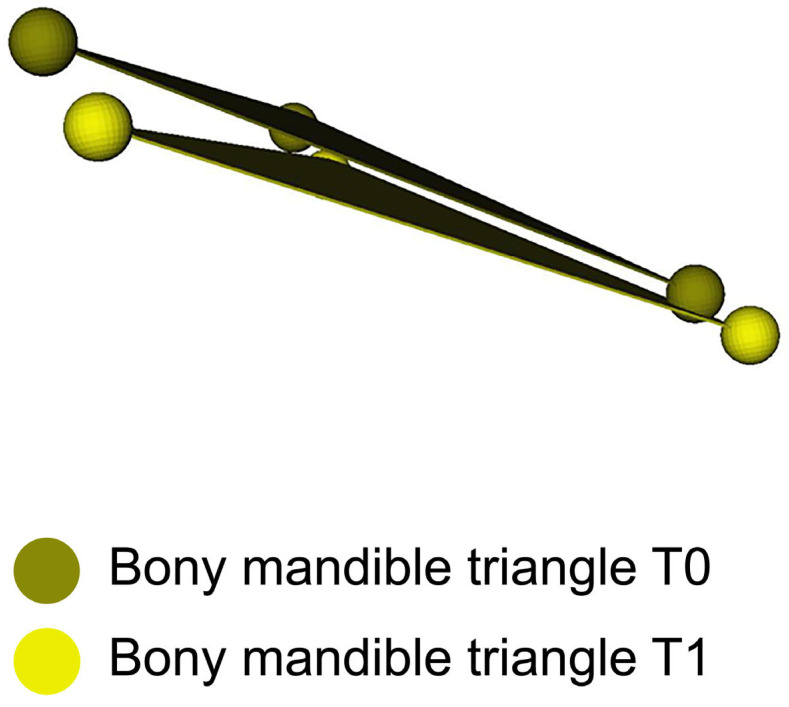
Example of triangles of T0 and T1. A Procrustes transformation was applied between triangles to align the pretreatment orthognathic planning dataset (created before orthodontic treatment) with the preoperative orthognathic planning dataset. This enabled calculation of translations and rotations of virtual triangles representing the maxillary and mandibular dental arches. The resulting transformation matrices captured positional and rotational changes of these structures from pretreatment to preoperative planning stages.

**Figure 8:**
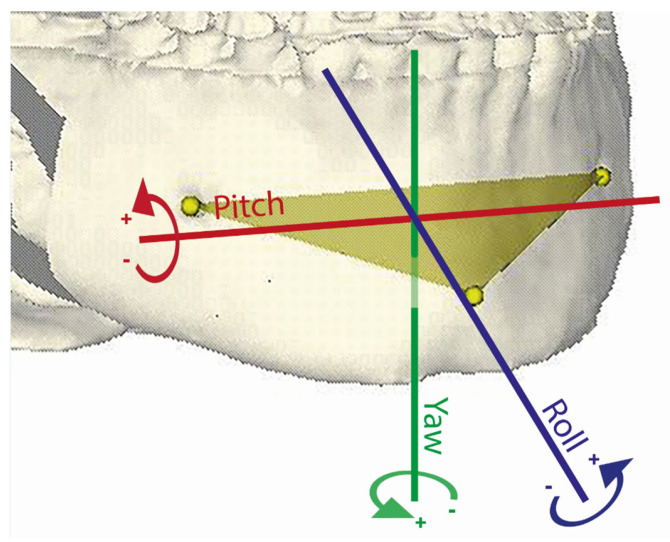
Visualization of rotational changes on mandibular bony triangle. Rotational changes are displayed as pitch (tilting up or down around the left–right axis), roll (tilting side-to-side around the front–back axis), and yaw (rotation left or right around the vertical axis).

A Procrustes transformation was applied to align the pretreatment orthognathic planning dataset (created prior to orthodontic intervention) with the preoperative orthognathic planning dataset, enabling calculation of the translations and rotations of the virtual triangles between the two datasets (Figure 7). The resulting transformation matrix captured details of the positional and rotational changes of the maxillary and mandibular dental arches from their pretreatment planning positions to the preoperative planning stage. Subsequently, the OrthoGnathicAnalyser tool in 3DMedX converted these transformation matrices into clinically meaningful data, including anterior–posterior, left–right, and vertical translations, as well as pitch, roll, and yaw movements for each dental arch or mandibular bony segment (Figure 8).

### 2.6. Statistical Analyses

All statistical analyses were conducted using R, version 4.1.3 (R Core Team, Vienna, Austria). One-sample *t*-test analyses were used to calculate the dental and bony mandibular differences between T0 and T1. To describe absolute differences, out-of-range values were used for translational and rotational differences when these differences exceed the clinical tolerance levels of 2 mm for translations and 4° for rotations as well as a top 10 percent value which means 10 percent of the differences were above or below this given value. Unpaired *t*-test analyses were used to perform analyses for SARME and extraction therapy.

## 3. Results

Notably, 26 patients were enrolled in this study: 17 females (65%) and 9 males (35%). The mean age at surgery was 30 (range 22–51) years. Notably, 6 patients were class III patients, and 20 patients had a class II malocclusion. The mean treatment time between the start of orthodontic treatment and surgery was 19.4 months (range 7–32 months). Moreover, 15 patients underwent a SARME procedure prior to the bimaxillary surgery; one patient had a Le Fort I osteotomy instead of a bimaxillary surgery. Notably, 13 patients received extractions to align the teeth; 12 had two premolar extractions in the mandible, one had two molar extractions in the mandible, and 4 had two premolar extractions in the maxilla in addition to lower premolar extractions.

### 3.1. T0 Planning Accuracy

To evaluate the accuracy of the dental aspect of T0 planning, the virtual triangles on the dental arches of the orthodontic setup in the T0 planning and the triangles on the dental arches in the T1 planning were compared. To evaluate the bony changes, the triangles on the mandibular distal segment were compared between T0 and T1 planning.

The values for each parameter are presented in Table 1. Roll refers to rotation around the sagittal axis, where a positive value indicates an anti-clockwise rotation about this axis. Pitch denotes rotation around the transverse axis, with a positive value representing an anti-clockwise rotation as viewed from the patient’s right side. Yaw describes rotation around the vertical axis, where a positive value indicates an anti-clockwise rotation (Figure 8).

The X-axis represents left-to-right displacement; a positive value indicates that the T1 planning is shifted further left relative to the T0 planning, while a negative value signifies a shift to the right. The Y-axis describes anterior-to-posterior movement; a positive value means the T1 planning is positioned more anteriorly than planned, whereas a negative value indicates a posterior displacement. The Z-axis reflects cranial-to-caudal movement, with positive values showing that the maxilla is positioned more cranially compared to the planned position.

Significant differences were found for the pitch of the maxillary dental arch with a mean difference of −2.85°, for the left/right (LR) translation of the mandibular dental arch with a mean value of 1.19 mm.

Table 2 shows the percentage of out-of-range differences for all parameters if clinical tolerance limits are set at 2 mm for translations and 4° for rotations. Relatively large differences were found for the pitch for rotations and the dental differences in the mandible.

### 3.2. Effect of SARME and Extraction on the Results

Table 3 shows the effect of SARME and extraction therapy between T0 and T1 on the accuracy of the planning. A significant difference was found for SARME patients for the bony mandible, with significantly larger differences for the rotations around the horizontal axis (roll) in an anti-clockwise direction. Significant differences are also found for the pitch in the dental maxilla and bony mandible with both relatively larger clockwise rotations around the transversal axis in maxillary extraction cases. Extraction therapy in the mandible had no significant effect on the planning accuracy.

## 4. Discussion

### 4.1. Interpretation of the Study Results

In this retrospective study, we assessed the accuracy of the pretreatment orthognathic planning at the start of presurgical orthodontic treatment in comparison to the actual orthognathic planning subsequent to orthodontic alignment. The results of the current study reveal noteworthy differences between the initial planning (T0) and subsequent planning (T1). Specifically, the T0 planning exhibited a more anti-clockwise rotation of the dental maxilla compared to the T1 planning. Importantly, some of these differences, particularly at the dental level, exceed clinically accepted thresholds, indicating limitations in the current workflow’s predictive accuracy. A plausible explanation for this difference lies in the unforeseen dental side effects resulting from the extraction procedure that were not entirely anticipated in the pretreatment setup. These side effects manifest as a more pronounced deepening of the curve of Spee and extrusion of the incisors, particularly notable in cases involving tooth extraction compared to nonextraction cases [22,23]. Among our study cohort, 15% underwent premolar extraction in the maxilla, and a potential effect is confirmed through subgroup analyses of the impact of premolar extraction in the maxilla, revealing a small but significant effect on pitch in the maxilla. For SARME patients, the dental mandible demonstrates a significant deviation to the right compared to the initial setup. Although no clear explanation for this observation can be provided based on our current data, possible contributing factors could include asymmetric expansion forces, responses to latent skeletal asymmetry becoming evident after expansion, and potential functional adaptations (e.g., habitual shifts or subtle condylar remodeling) that may contribute to observed deviations [24]. Furthermore, only 3 significant values in 54 tests for SARME and extraction patients suggest this may be a chance finding, so conclusions should be drawn with caution.

The method was previously validated in a study conducted by Baan et al. [25]. The 3DmedX software, used for registration of the dental and bony segments and calculation of the result, is user-friendly. The software is designed with ease of use in mind, providing an intuitive and accessible interface for clinicians. It emphasizes a streamlined workflow, which can be built by the user to address all needs, shortening the learning curve and enhancing its practical application.

Regarding method errors, that study reported minimal intra-observer and inter-observer variations in measurement errors (mean error < 0.25 mm) and high intraclass correlation coefficients (>0.97). These findings support the observer-independent nature of the methodology. Notably, the pitch rotations of the maxilla and mandible in the earlier-mentioned study [25] showed the most significant measurement discrepancy, measuring 2.72° and 2.75°, respectively. This observation could be an explanation for the differences found for pitch rotations in the current study.

### 4.2. Clinical Relevance

To assess the clinical significance of discrepancies between the planned setup and the surgical outcome, tolerance thresholds were applied. Drawing from values reported in the literature [26], cutoff limits were set at 2 mm for translational differences and 4 degrees for rotational deviations. In the dental maxilla, 15.39% of rotational mean differences and 21.80% of translational mean differences exceeded these thresholds (Table 2). For the dental mandible, 33.33% of rotational mean differences and 44.87% of translational mean differences fell outside the clinically acceptable range. Similarly, in the bony mandible, 15.38% of rotational mean differences and 17.95% of translational mean differences surpassed the established clinical limits (Table 2). Despite significant disparities found between the dental setup and outcome at the T1 planning, these discrepancies did not result in notable mean differences at the bony planning level except for the pitch in the bony mandible in maxillary extraction cases. However, the high percentages of out-of-range differences are clinically relevant. These findings imply that the method may not yet be sufficiently robust for clinical application. Future investigations should prioritize improving the predictability of orthodontic preparations to enhance the accuracy of planning methods. Our results indicate that improvements should focus on achieving the correct torque and vertical position of the upper anterior teeth. Intra-oral scans taken at consecutive time points during the orthodontic preparation phase could be useful for tracking progress against the original plan, helping to ensure a more predictable outcome. Furthermore, other potential measures to improve the reliability of the workflow include improving the accuracy of intra-oral scanning and digital registration, and developing predictive algorithms to better anticipate orthodontic tooth movement.

### 4.3. Limitations in Study Design

The methodology employed in this study did not encompass soft tissue assessment, an essential component in orthognathic treatment planning. Further research should address whether these differences could significantly affect the soft tissue outcomes resulting from the planning. In our sample, only one patient had SARME plus maxillary extractions. Therefore, it was not possible to perform a statistical analysis for the combined effect of extractions and SARME. An important limitation of the present study is the inclusion of a relatively small and heterogeneous sample comprising both Class II and Class III malocclusion cases. These groups have fundamentally different orthodontic treatment objectives, particularly regarding incisor decompensation and surgical planning. Analyzing them as a single cohort may have introduced variability and reduced the precision of our findings. Although the combined analysis offers a broader initial insight into the feasibility of digital setups across different malocclusion types, future studies should focus on larger and more homogeneous cohorts or perform separate analyses for Class II and Class III patients. This would allow for more precise evaluation of how specific skeletal patterns and treatment mechanics influence the accuracy of digital planning and surgical outcomes. Another limitation of our study involves the unexplored variations for the bony maxilla, because it served as a reference point for evaluating the parameters. To enhance our understanding, further research should specifically address and investigate these differences for the bony maxilla.

Sole reliance on a single operator for fabricating the orthodontic setups introduces certain limitations. While this approach was intended to eliminate inter-operator variability, it may instead introduce single-operator bias, potentially limiting the generalizability of the findings. Future research should consider involving multiple operators or developing automated setup generation using artificial intelligence tools to better assess inter-operator variability and improve the external validity of the results. The method is susceptible to inter-operator variability. Previous evidence indicates that inter-operator differences in producing setups were clinically acceptable for 74% to 90% of the rotational and translational differences between setups of different operators [27]. The variability among clinicians involved in orthodontic treatment represents another limitation. Although all patients had their treatment at the university clinic, potential clinician bias cannot be disregarded.

An additional limitation of the present study is the complexity of the proposed digital workflow, which requires multiple software platforms. While this multi-software approach allows for advanced analyses and precise virtual planning, it also introduces practical barriers to widespread clinical adoption due to the need for significant expertise, time investment, and resource availability. Furthermore, the involvement of multiple software systems increases the risk of cumulative errors arising from data transfers and interoperability issues. Future developments should aim to integrate these processes into more streamlined, user-friendly platforms to enhance clinical feasibility and minimize potential sources of error.

## 5. Conclusions

The following conclusions can be drawn based on our results and the limitations in study design:

- The accuracy of the presented orthognathic surgical planning workflow prior to orthodontic intervention is limited at the dental level, particularly regarding the torque of the maxillary anterior teeth.
- No significant differences were observed between pretreatment planning and actual orthodontic planning at the level of the bony mandible; however, a noteworthy proportion of rotational and translational discrepancies exceeded clinically acceptable thresholds.
- Although the proposed method demonstrates potential as a simulation tool for predicting mandibular bony outcomes at the start of treatment, the current accuracy is insufficient for reliable routine clinical use. Further refinement and validation in larger, more homogeneous patient groups are necessary to improve its clinical applicability.

## Figures and Tables

**Table 1:** Mean differences for dental maxilla (A), dental mandible (B), and bony mandible (C) between virtual triangle in T0 planning versus virtual triangle in T1 planning. Mean differences, standard error of the mean (SEM), 10% of the observations above this absolute value (top-10p) above this value, 95% confidence interval (CI), and results of one-sample *t*-test analyses (*p*-value).

	Mean Differences	SEM	Top 10% Value	95% CI		*p*-Value
Dental maxilla						
Pitch ^a^	−2.85	0.68	7.47	−4.25	−1.44	**<0.001**
Roll ^b^	0.05	0.21	1.47	−0.39	0.49	0.804
Yaw ^c^	−0.54	0.33	2.19	−1.23	0.14	0.114
X (LR) ^d^	0.34	0.21	1.75	−0.10	0.79	0.120
Y (AP) ^e^	−0.74	0.54	4.44	−1.84	0.37	0.181
Z (UD) ^f^	−0.82	0.41	2.89	−0.03	1.67	0.059
Dental mandible						
Pitch	−1.63	1.30	9.75	−4.31	1.05	0.222
Roll	0.51	0.72	5.51	−0.98	2.00	0.485
Yaw	−1.52	0.92	7.54	−3.41	0.38	0.111
X (LR)	1.19	0.51	4.49	0.15	2.24	**0.027**
Y (AP)	0.72	0.45	3.70	−0.21	1.64	0.124
Z (UD)	0.02	0.51	3.88	−1.03	1.07	0.970
Bony mandible						
Pitch	−0.41	0.71	5.88	−1.87	1.05	0.566
Roll	0.31	0.39	2.56	−0.49	1.11	0.430
Yaw	−1.21	0.49	5.06	−2.23	−0.20	0.121
X (LR)	0.76	0.37	3.45	0.00	1.52	0.052
Y (AP)	0.12	0.28	2.38	−0.46	0.70	0.674
Z (UD)	0.08	0.27	1.99	−0.48	0.63	0.777

Statistically significant values are marked in bold. Rotational movements are in degrees and translational movements in mm. Significance was set a α = 0.05. ^a^ Pitch: Positive values denote an anti-clockwise rotation relative to the T0 planning, while negative values indicate a clockwise rotation around the transverse axis compared to T0. ^b^ Roll: A positive value reflects an anti-clockwise rotation around the horizontal axis relative to T0, whereas a negative value signifies a clockwise rotation. ^c^ Yaw: Positive values correspond to an anti-clockwise rotation around the vertical axis relative to T0, and negative values indicate a clockwise rotation. ^d^ Translation left right (LR): A positive value means the T1 planning is shifted further to the left compared to T0; a negative value indicates a shift to the right. ^e^ Translation anterior posterior (AP): Positive values indicate that T1 is positioned more anteriorly than planned, while negative values reflect a posterior shift. ^f^ Translation upward downward (UD): A positive value shows cranial (upward) displacement of the maxilla relative to the plan, while a negative value signifies a caudal (downward) shift.

**Table 2:** Out-of-range differences (percentages) for the dental maxilla, dental mandible, and bony mandible. Cutoff limits are set at 2 mm for translations and 4° for rotations.

	Percentage out of Range (%)		
Parameter	Dental Maxilla	Dental Mandible	Bony Mandible
Pitch	42.31	57.69	26.92
Roll	0.00	15.38	3.85
Yaw	3.85	26.92	15.38
Mean rotations	15.39	33.33	15.38
Trans LR	11.54	50.00	26.92
Trans FB	23.08	50.00	15.38
Trans UD	30.77	34.62	11.54
Mean translations	21.80	44.87	17.95

**Table 3:** Effect of SARME and extraction therapy on the differences between T0 planning and T1 planning, mean difference between patients with and without effect per jaw, 95% confidence interval (CI), and results of the unpaired *t*-test (*p*-value) per parameter.

	Dental Maxilla				Dental Mandible				Bony Mandible			
Parameter	Mean Diff	*p*	95% CI		Mean Diff	*p*	95% CI		Mean Diff	*p*	95% CI	
Effect of SARME (*n* = 15)												
Pitch	−2.39	0.084	−5.13	0.35	−3.15	0.247	−8.62	2.34	−1.33	0.358	−4.27	1.62
Roll	−0.10	0.826	−0.99	0.80	2.23	0.100	−0.46	4.92	1.58	**0.028**	0.18	2.98
Yaw	0.72	0.311	−0.73	2.18	−2.08	0.280	−5.98	1.82	−1.35	0.199	−3.46	0.77
Trans LR	−0.39	0.381	−1.30	0.52	0.89	0.378	−1.16	2.94	0.97	0.191	−0.52	2.47
Trans FB	−0.02	0.980	−2.10	2.05	0.43	0.630	−1.38	2.23	0.46	0.413	−0.68	1.59
Trans UD	−0.20	0.798	−1.81	1.41	0.17	0.866	−1.89	2.23	0.62	0.263	−0.50	1.73
Effect extraction in the maxilla (*n* = 4)												
Pitch	−8.30	**0.020**	−14.71	−1.89	−1.98	0.409	−8.02	4.06	−3.57	**0.038**	−6.88	−0.27
Roll	−0.23	0.543	−1.05	0.59	−2.69	0.164	−6.94	1.55	−1.29	0.110	−2.96	0.37
Yaw	0.27	0.760	−1.96	2.51	−0.24	0.852	−2.93	2.44	−0.54	0.464	−2.07	0.98
Trans LR	0.13	0.763	−0.89	1.16	0.37	0.678	−1.58	2.32	−0.11	0.871	−1.63	1.40
Trans FB	−0.29	0.845	−3.94	3.37	−0.20	0.809	−1.99	1.59	−0.19	0.729	−1.45	1.07
Trans UD	0.32	0.733	−1.87	2.51	−0.18	0.897	−3.70	3.34	−0.15	0.813	−1.67	1.37
Effect extraction in the mandible (*n* = 13)												
Pitch	−0.480	0.733	−3.364	2.404	−1.18	0.501	−4.79	2.41	−1.97	0.168	−4.84	0.89
Roll	−0.453	0.297	−1.331	0.425	−0.65	0.412	−2.24	0.95	−0.05	0.947	−1.70	1.59
Yaw	0.356	0.604	−1.046	1.758	−1.12	0.300	−3.32	1.07	−1.61	0.103	−3.57	0.35
Trans LR	−0.628	0.146	−1.491	0.235	−0.20	0.846	−2.34	1.94	0.16	0.834	−1.40	1.71
Trans FB	−1.053	0.339	−3.308	1.202	−0.29	0.754	−2.18	1.60	−0.26	0.659	−1.44	0.93
Trans UD	−0.122	0.886	−1.878	1.634	−1.51	0.141	−3.56	0.54	−0.20	0.717	−1.35	0.94

Statistically significant values are marked in bold. Rotational movements are in degrees and translational movements are in mm. Significance was set a α = 0.05.

## Data Availability

The raw data supporting the conclusions of this article will be made available by the authors on request.

## Abbreviations

The following abbreviations are used in this manuscript:

CBCT	Cone Beam Computed Tomography
3D	Three-Dimensional
STL	Standard Tessellation Language
DICOM	Digital Imaging and Communications in Medicine
VBM	Voxel-based matching
SARME	Surgically Assisted Maxillary Expansion
UD	Upward Downward
LR	Left Right
AP	Anterior Posterior
